# An alternative palliative surgical method for advanced malignant obstructive jaundice: Laparoscopic bridge choledochoduodenostomy

**DOI:** 10.3389/fsurg.2022.1056093

**Published:** 2023-01-06

**Authors:** Tao Lianyuan, Xiao Hongsheng, Zou Xuxiang, Wang Liancai, Lei Dazhao, Li Deyu

**Affiliations:** ^1^ Department of Hepatobiliary Surgery, Henan Provincial People’s Hospital, People’s Hospital of Zhengzhou University, People’s Hospital of Henan University, Zhengzhou, China; ^2^ Department of General Surgery, Central Hospital of Dengzhou, Dengzhou, China

**Keywords:** obstructive jaundice, choledochoduodenostomy, laparoscopic, biliary drainage, bilioenteric anastomosis

## Abstract

**Background:**

This study introduces an alternative palliative surgical procedure called laparoscopic bridge choledochoduodenostomy (LBCDD) for patients with advanced malignant obstructive jaundice (AMOJ).

**Methods:**

Patients with AMOJ who had LBCDD between January 2017 and August 2021 were identified from databases of two institutions in China.

**Results:**

A total of 35 patients (male 12; female 23) with an average age of 64 years were enrolled. The average diameter of the tumor is 4.24 cm. All patients undertook LBCDD within an average operation time of 75 min with a mean blood loss of 32 ml. One patient had controlled bile leakage after the operation and two developed surgical site infection involving the epigastric orifices. All of them were solved by conservative treatment. All patients were discharged smoothly after an average hospital stay of 5.5 days, and no conversion to open surgery was required.

**Conclusions:**

LBCDD is a safe and efficient palliative surgery, which has a good therapeutic effect on patients with AMOJ.

## Introduction

Malignant obstructive jaundice can cause many adverse events including severe cholangitis, lower the quality of life, and increase mortality, which can occur following pancreatic cancer, hilar cholangiocarcinoma, and periampullary carcinoma (1–4). For advanced malignant obstructive jaundice (AMOJ) with no chance for radical cure, although combined treatment and local treatment are indispensable (5–7), effective and reliable biliary drainage is the most important palliative treatment (1–4, 8, 9). Percutaneous transhepatic biliary drainage (PTBD), endoscopic biliary drainage (EBD) and bilioenteric anastomosis are the commonly used clinical methods for AMOJ at present (2, 9, 10). As an external biliary drainage, PTBD may lead to nutritional loss, gastrointestinal dysfunction, and a series of stable immune systems due to the long-term loss of large amounts of bile (3, 9, 11–13). Moreover, with low compliance, tube outside the body may cause psychological burden (3, 9, 11). As internal biliary drainage, EBD and bilioenteric anastomosis can avoid external biliary drainage problems. However, EBD cannot be applied to cases of severe biliary obstruction (4, 9, 11). Bilioenteric anastomosis is considered to be the most effective palliative treatment for advanced malignant obstructive jaundice. However, for malignant cases involving high bile duct position, some patients cannot complete bilioenteric anastomosis because of the short normal bile duct, such as advanced hilar cholangiocarcinoma (8, 14, 15). Therefore, we present a new laparoscopic surgical procedure, which bridges the common bile duct and duodenum through a T-tube and constructs a bile internal drainage. The new surgical procedure was called laparoscopic bridge choledochoduodenostomy (LBCDD), as the T-tube acted as a bridge for bile drainage in this surgical procedure. This surgical method may provide an alternative way of internal bile drainage for AMOJ. The present study is to assess the efficacy, safety, and feasibility of this novel surgical procedure.

## Methods

### General information and grouping

Patients with AMOJ who had LBCDD between January 2017 and August 2021 were identified from the electronic database of Central Hospital of Dengzhou and Henan Provincial People's Hospital. Inclusion criteria included patients with obstructive jaundice due to bile duct and pancreatic, ampullary, or duodenal malignancy who had lost the opportunity for radical or transformational therapy. The current treatment for these patients is mainly to relieve jaundice, and the patients or their family members refused external drainage and strongly required internal drainage. The present study was approved by the ethics committee of the hospitals. All patients signed the informed consent. A total of 35 patients with AMOJ who had LBCDD were enrolled. Fifteen cases of pancreatic carcinoma, 12 cases of terminal bile duct carcinoma, 5 cases of ampullary carcinoma, and 3 cases of duodenal adenocarcinoma were involved in this study. All diagnosis was confirmed by B-ultrasound, computed tomography (CT), magnetic resonance imaging (MRI), or magnetic resonance cholangiopancreatography (MRCP). Among them, 21 patients also had preoperative endoscopic procedures. Because most of the patients did not receive tumor resection or puncture biopsy, their diagnosis was clinical diagnosis without histopathological confirmation. Each operation was evaluated repeatedly on the basis of preoperative data and intraoperative situations under laparoscopy to ensure surgical safety. All patients had a good clinical record and were identified as being in an advanced stage, losing the chance of radical surgery. Recorded data such as symptoms, comorbidities, blood imaging studies, investigations, surgical data, postoperative variables, and follow-up data were collected. Continuous variables are represented by median values. The Charlson Comorbidity Index (CCI) was used to define the severity of comorbid conditions. Since patients’ readmission to hospital may be strongly influenced by factors other than their condition, we did not count readmissions for evaluation.

### Positions of trocars and trimming of T-tube of LBCDD

**Figure 1:**
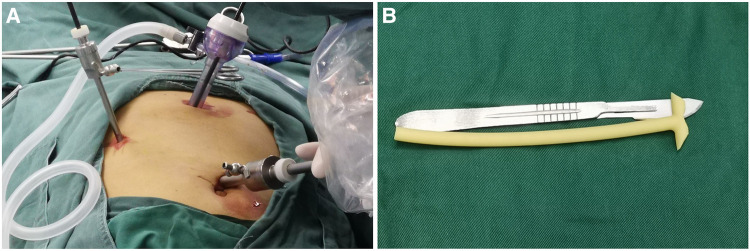
Positions of trocars and trimming of T-tube. Patients were placed in the supine position or slightly inclined to the left on the operation table. After general anesthesia and intubation, the operating area was then disinfected. A 10 mm incision was first made at the right edge of the umbilicus and a 10 mm trocar was placed. Then, the laparoscope was placed after the pneumoperitoneum was constructed. Under the guidance of the laparoscope, two trocars for surgical instruments were placed below the xiphoid process (10 or 12 mm) and 2 cm below the costal margin of the right upper quadrant along the median line of the clavicle (5 mm), respectively (**A**). If assistance is required, a trocar (5 mm) can also be placed along the midline of the clavicle 2 cm below the costal margin of the right upper quadrant. The T-tube serves as a bridge for bile from the common bile duct into the duodenum, retaining a length of 10–12 cm to ensure that the distal T-tube of the duodenum can pass through the duodenal papilla (**B**).

Patients were placed in supine position or slightly inclined to the left on the operation table. After performing general anesthesia and intubation, the operating area was then disinfected. A 10 mm incision was first made at the right edge of the umbilicus and a 10 mm trocar was placed. Then, the laparoscope was placed after the pneumoperitoneum was constructed. Under the guidance of the laparoscope, two trocars for surgical instruments were placed below the xiphoid process (10 or 12 mm) and 2 cm below the costal margin of the right upper quadrant along the median line of the clavicle (5 mm), respectively (Figure 1A). If assistance is required, a trocar (5 mm) can also be placed along the midline of the clavicle 2 cm below the costal margin of the right upper quadrant. The T-tube serves as a bridge for bile from the common bile duct into the duodenum, retaining a length of 10–12 cm to ensure that the distal T-tube of the duodenum can pass through the duodenal papilla (Figure 1B).

### Surgical procedure of LBCDD and relevant precautions

**Figure 2:**
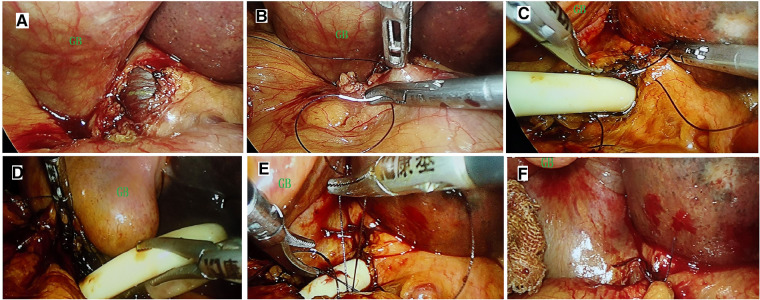
Surgical procedure of laparoscopic bridge choledochoduodenostomy. After the exploration of the abdominal cavity, the common bile duct was exposed first (**A**). An ostomy was made in the duodenum below the common bile duct, and a presutured double-layer suture was performed around (**B**). The trimmed T-tube was placed in the common bile duct and fixed (**C**). After the distal end of the T-tube was placed in duodenum through the ostomy (**D**), the presutured double-layer suture line was tightened (**E**). Then the adjacent greater omentum tissue was pulled to cover the T-tube (**F**). GB, gall bladder.

After the exploration of the abdominal cavity, the common bile duct was exposed first (Figure 2A). An opening was made in the duodenum below the common bile duct, and a presutured double-layer suture was performed around (Figure 2B). The trimmed T-tube was placed in the common bile duct and fixed (Figure 2C). After the distal end of the T-tube was placed in duodenum through the open (Figure 2D), the presutured double-layer suture line was tightened (Figure 2E). Then, the adjacent greater omentum tissue was pulled to cover the T-tube (Figure 2F).

The surgical procedure can be simplified into three steps. The first step is to place a trimmed T-tube into the common bile duct and suture it for fixation. The second step is to place the distal end of the T-tube into the duodenal open and suture it for fixation. Then, adjacent tissues such as the greater omentum can be used to cover the exposed portion of the T-tube. It should be emphasized that the opening of the bile duct and duodenum should be as close together as possible and that the length of the T-tube in the duodenal lumen should extend beyond the duodenal papilla.

During the operation, to prevent bile or intestinal contents leaking into the abdominal cavity from the cutting open, we usually put an aspirator in the precut open position before incision, which can suck up the leaked bile or intestinal contents and minimize the abdominal pollution. Prophylactic application of the second-generation cephalosporin was applied for 24 h postoperatively, which could be extended to 48 h for individual patients according to intraoperative conditions.

### Statistical analysis and follow-up

The cumulative summation (CUSUM) test was applied for the quantitative estimation of the learning curve (plotting the operation time and blood loss, and determination of the case number to achieve mastery) as described (16). Continuous variables were presented as mean ± SD and mean (range). Follow-up was performed by trained investigators through telephone calls, by recording the consultations of patients at the outpatient clinic every 2 weeks for 2 months postoperatively.

## Results

A total of 35 patients were enrolled, including 12 males and 23 females with a mean age of 64 (±10.65) years. The average body mass index of all patients was 26.15 (±3.5). The average diameter of the tumor is 4.24 (±1.11) cm, with a minimum of 3.5 cm and a maximum of 9.5 cm. Among them, 27 patients have a diameter of over 4 cm. All operations were performed within an average operating time of 75 (45–120) min with a mean blood loss of 32 (5–150) ml. The range of preoperative total bilirubin of all patients was between 135.1–632.5 mol/L, with a mean value of 241.24 ± 101.55 mol/L. Patients who developed comorbidities were kept in the ICU for 1 day after the operation. There was one patient who developed a controlled bile leak and two had surgical site infection (SSI) involving the epigastric port. All of them were resolved through a conservative way. The drain tube was removed 3 days postoperatively after a routine abdominal imaging examination, except the cases who had bile leak. There are no postoperative mortalities. All the patients were discharged smoothly with a mean hospital stay of 5.5 days, and no conversion to open surgery was required. During the mean follow-up duration of 14 (±4.3) months, no anastomose-related long-term complications have been found, which include strictures, cholangitis, or pancreatitis (Table 1). After operation, 29 patients received further chemotherapy and 8 accepted radiotherapy. By the end of December 2021, 29 patients had died, of which 1 patient died of gastrointestinal bleeding, and the others died of malignant fluid and systemic failure caused by the tumor. The median survival was 8.2 (±4.1) months. All patients were followed up and the results showed that total bilirubin had fallen below 50 mol/L in all patients 2 weeks after surgery.

**Table 1:** Patient and surgery characteristics.

Variable value	Value (mean ± SD)
Age (years)	64 ± 10.65
Sex	
Male	12 (34.3%)
Female	23 (65.7%)
Diagnosis (cases)	
Pancreatic carcinoma	15 (42.86%)
Terminal bile duct carcinoma	12 (34.29%)
Ampullary carcinoma	5 (14.29%)
Duodenal adenocarcinoma	3 (8.57%)
Body mass index (kg/m^2^)	26.15 ± 3.5
CBD diameter (cm)	1.5 ± 0.7
Total bilirubin (mol/L)	241.24 ± 101.55
ALT (U/L)	86 ± 107
Tumor diameter (cm)	4.24 ± 1.11
AJCC stage	
III	16 (45.9%)
IV	19 (54.1%)
Charlson comorbidity index	
0	27 (77.1%)
1–3	8 (22.9%)
Operative time (min)	75 (45–120)
Blood loss (ml)	32.0 (5–150)
Complication	3 (8.6%)
Bile leak	1 (2.9%)
SSI-superficial	2 (5.7%)
Hospital stays (days)	5.5 ± 2.5

SD, standard deviation; CBD, common bile duct; SSI, surgical site infection.

Based on a visual analysis of the learning curve, a peak was noted in the 13th case (detailed information is listed in the Supplementary Material). Therefore, case 13 was defined as the learning-curve cutoff point regarding surgical time, blood loss, and complications after which the learning curve declined.

## Discussion

Our study showed that the majority of AMOJ patients were elderly (64 ± 10.65 years), and females were 1.92 times as many as males. Since there was no opportunity of radical surgery for AMOJ patients, solving jaundice was the most important way to prolong life and improve their life quality. Most of the patients have a large tumor above 4 cm, which severely compacts or infiltrates the bile duct, making EBD impossible to perform. Bilioenteric anastomosis is reported including choledoduodenostomy and choledojejunostomy. This procedure could not be performed in patients enrolled in this study, mainly because the high bile duct was invaded by the tumor, and there was no sufficient length of normal bile duct for the anastomosis (17–19). Moreover, bilioenteric anastomosis has the risk of complications such as anastomotic leak and strictures (14, 15, 19, 20).

LBCDD is a novel internal drainage procedure, which avoids a series of external drainage-related complications such as weakened immunity and impaired gastrointestinal function caused by chronic and massive bile loss. In addition, with a high degree of compliance, LBCDD does not need to wear any tubes outside the body. LBCDD applied T-tube to drainage bile from common bile duct to the duodenum. A T was tube used as a bridge, which establish a channel between bile duct and duodenum. The T-tube length was controlled in 10–12 cm, so as to cross the duodenal papilla, which ensures that various digestive enzymes are activated away from the duodenal opening. Moreover, we used the greater omentum to cover the T-tube between the bile duct and duodenum. Those measures have effectively reduced the risk of anastomotic leaks. Duodenal leak is considered dreaded when we begin the procedure; however, none of the cases had this complication. Although the published leak rate of choledojejunostomy is 2%–7% (14, 15, 17), there is only one case of biliary leakage. In addition, there are two cases of surgical site infection. All of them occurred in the early stages of our learning curve. According to the follow-up data, the total bilirubin of all patients had fallen below 50 mol/L 2 weeks after surgery, and no delayed postoperative complications such as cholangitis, pancreatitis, and strictures occurred. Therefore, for patients with AMOJ who cannot be treated with EBD or bilioenteric anastomosis, as a safe surgical procedure, LBCDD may be an alternative for internal bile drainage.

In addition, the operation process of the present operation is simple and the operation time is short. Most of them can be completed around 1 h in the later stage of the term curve (after the learning-curve cutoff point of the 13th case), with an average operative time of 75 (±31) min. On one hand, the simplified surgical procedures can reduce the complications related to the operation. On the other hand, it also can reduce the operation cost and speed up postoperative recovery. The patient can have a liquid diet on the second day after the operation. Early eating can improve patient's in-patient experience and satisfaction, as well as ensure the patient's smooth postoperative recovery. The current study reported a comparable short hospital stay with a median length of 5.5 days.

Our study shows that LBCDD, as a novel surgical procedure, is a safe and efficient treatment for AMOJ. Compared with bilioenteric anastomosis, LBCDD does not need to cut the small intestine; it has a simpler surgical procedure, with less bleeding risk, requires no expensive supplies, and is more physiological. Therefore, LBCDD is worthy of recommendation. Since our study enrolled only 35 patients, the number is small, and the implementation of this technique requires sophisticated laparoscopic techniques; the replication of similar results may not be achieved during the early stages of performing this procedure. Moreover, this procedure requires an opening in the duodenum, there is a theoretical possibility of duodenal leakage for inexperienced physicians or patients with poor postoperative management. However, avoiding a series of external drainage-related shortcomings and with a high degree of compliance, LBCDD is a safe and simple operation, which can reduce the operation cost and speed up postoperative recovery. We would like to suggest LBCDD as an alternative option.

## Conclusion

LBCDD is a safe and efficient palliative surgery, which has a good therapeutic effect on patients with AMOJ.

## Data Availability

The raw data supporting the conclusions of this article will be made available by the authors, without undue reservation.
