# Supplementary material for: An alternative palliative surgical method for advanced malignant obstructive jaundice: Laparoscopic bridge choledochoduodenostomy
Source: Front Surg. 2023 Jan 6;9:1056093. doi: 10.3389/fsurg.2022.1056093 (PMC9852326; doi:10.3389/fsurg.2022.1056093)
Supplement: Supplementary file 1 [file Datasheet1.pdf]

Supplementary Material

The calculation formula of learning curve evaluation index is: $\alpha = Xi-Xo$, α is the quantitative value of the surgical index, Xo is the probability that the evaluation index fails to reach the target value, and Xi represents whether each operation reaches the target value. When the evaluation index reaches the target value, Xi=0, and if the target value is not reached, Xi=1.

Operation time(α1): The average operation time was 75 min (target value). If the target value is reached by 23%, it will be 77%, α 1=Xi-0.77. If the operation time in the study group is ≤ 75 min, then α1=0-0.77=-0.77; Operation time > 75 min, then α 1=1-0.77=0.23.

Intraoperative bleeding(α2): The average intraoperative bleeding volume was 32mL (target value). If the target value is reached by 31%, it will be 69%, α 2=Xi-0.69. In the study group, the amount of intraoperative bleeding is ≤ 32, then α 2=0-0.69=-0.69; If the intraoperative blood loss is more than 32mL. α2=1-0.69=0.31.

Complications (α3): The incidence of intraoperative and postoperative complications in the standard group was 9%. Therefore, it was 0.09%, α3=Xi-0.09. If there is no complication α3=0-0.09=-0.09; If complications occur α3=1-0.09=0.91.

Calculate the cumulative sum: Calculate the cumulative sum of the four evaluation indicators of all subjects, that is, the quantitative value of each operation, Σ= a1+ α 2+ α 3+ α 4. Then draw the learning curve based on summation by accumulation (Σ).

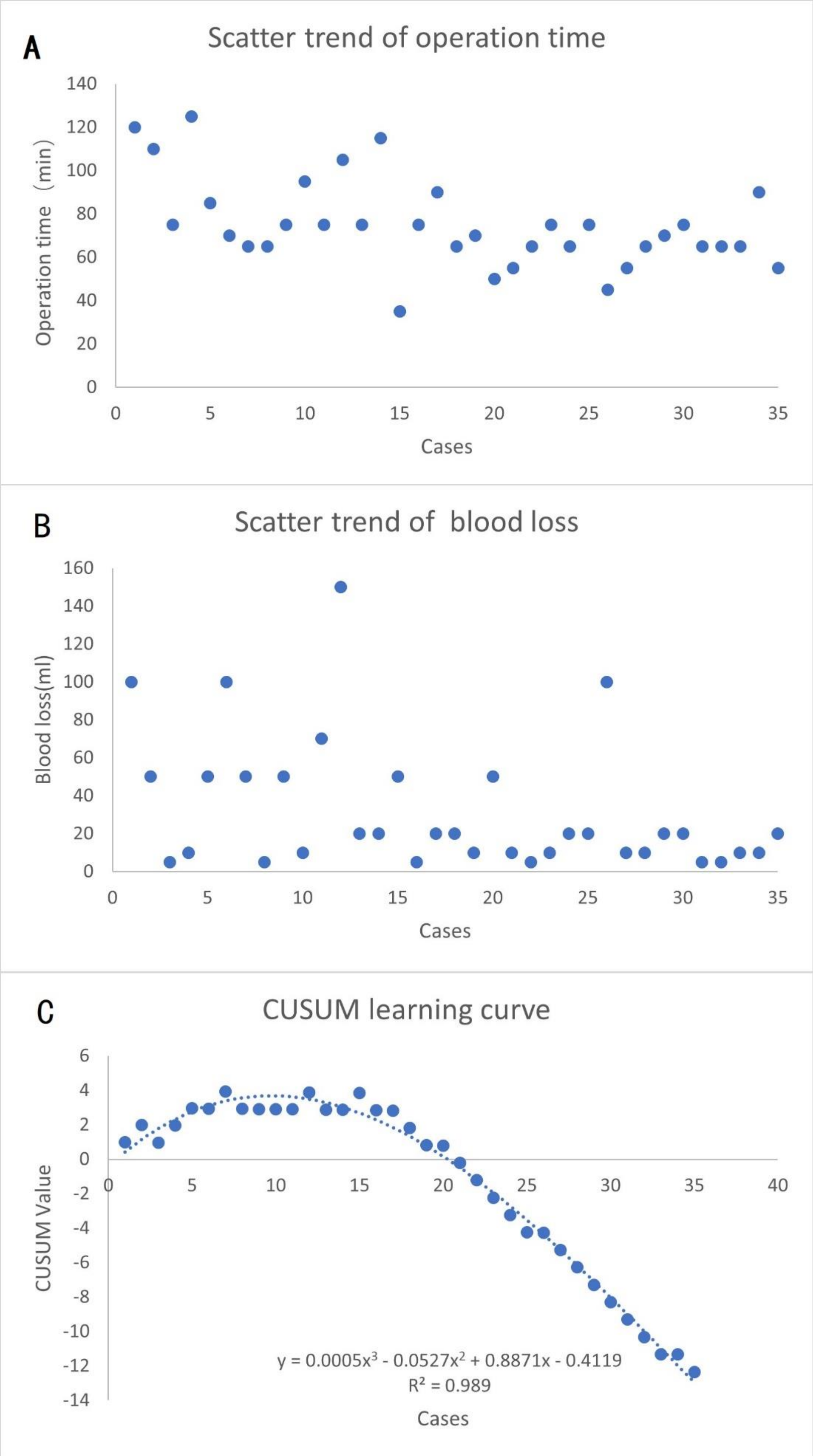

Supplementary figure 1. Learning curve studies of LBCDD. Operation time and blood loss plotted in chronological order (A, B), cumulative summation (CUSUM) test in the

patients who underwent LBCDD (C). The CUSUM plot decreased when the operative time exceeded this value. Based on a visual analysis of the learning curve, a peak was noted in the 13th case; therefore, case 13 was defined as the learning-curve cutoff point regarding surgical time, after which the learning curve declined.
